# Asylums in Politics

**Published:** 1895-03

**Authors:** 


Asylums in Politics.—Dr. James Orr touches this subject in plain United States language in a paper in this issue. Read it. The folly of disregarding the plain letter of the law in relation to the appointment of superintendents, has been clearly demonstrated under two administrations, and the unfortunate results of attempted administration of such institutions by novices, should serve as object lessons, and warn future governors against a repetition of it. But precedent is powerful in law, and justifies (?) many things.

There is much truth and force in what Dr. Orr says; but medical sentiment has been, tardily, it is true, aroused on the subject, and somebody has forestalled Dr. Orr's complaint in a measure, by getting before the legislature an act which, if it becomes a law, will, it is believed, remedy the evil complained of as regards the appointments. It repeals the law now in force, does away with the board of managers, or, rather, takes away the appointing power (they have not had the selective power for eight years or more, and their power to appoint has been limited to appointing the governor's selection), and puts it straight into the governor's hands, but it specifies in unmistakable language that the appointee shall have had not less than two years active experience in the treatment of the insane in an asylum.

It has been objected that there are few, very few physicians in Texas who will in future be eligible, under a strict construction of this act. But it can be unswered by the fact that during each administration there will be graduated and made available five physicians in the persons of the five assistant superintendents, two at Austin, two at Terrell, and one at San Antonio. The hope and immediate prospect of promotion to the superintendent's place will serve as additional stimulus to these young men to study and qualify themselves.

This bill has passed the senate, and at this writing is before the house committee. It is thought that it will become a law. If so, it will remove asylum superintendents from the list of political offices, which constitute the power of patronage. Dr. Orr will have more to say on the subject later, he says, especially if any one replies to his paper. The JOURNAL invites discussion of the subject.

